# Malaria exposure history shapes PD-1 expression across human B-cell subsets during acute *Plasmodium falciparum* infection

**DOI:** 10.1186/s12866-026-05388-8

**Published:** 2026-07-15

**Authors:** Susanne E Mortazavi, Allan Lugaajju, Muyideen Kolapo Tijani, Bingyan Wu, Lena Danielsson, Hans Norrgren, Kristina E M Persson

**Affiliations:** 1 Department of Laboratory Medicine, Lund University, Lund, Sweden; 2 Department of Infectious Diseases, Skåne University Hospital, Lund, Sweden; 3 College of Health Sciences, Makerere University, Kampala, Uganda; 4 Department of Clinical Sciences Lund, Lund University, Lund, Sweden; 5 Clinical Chemistry and Pharmacology, Laboratory Medicine, Skåne University Hospital, Region Skåne, Lund, Sweden

**Keywords:** Malaria, B cells, PD-1, Immune regulation, Antigen-specific immunity, Malaria exposure

## Abstract

**Background:**

Repeated malaria exposure shapes humoral immunity in endemic regions. Programmed cell death protein 1 (PD-1) is an inhibitory immune receptor involved in immune regulation during infection, but its expression across human B-cell subsets during acute malaria, and its relationship to exposure history and antigen specificity, remain poorly defined.

**Methods:**

PD-1 expression and circulating B-cell subsets were analyzed by flow cytometry in 64 individuals from Uganda and Sweden with different malaria exposure backgrounds, including individuals with acute malaria and healthy controls. PD-1 expression was assessed across major B-cell subsets and compared between *P. falciparum*-binding and non-binding B cells. Associations with parasitemia and inflammatory markers were also examined.

**Results:**

PD-1 expression was higher across both naïve and memory B-cell subsets during acute malaria in Ugandan individuals with ongoing malaria exposure compared with individuals with imported malaria diagnosed in Sweden. Within memory B-cell populations, PD-1 frequencies were highest on non-class-switched (IgD⁺ and IgM⁺) subsets and on *P. falciparum*-binding B cells. No differences in PD-1 expression were observed between paired acute and convalescent samples. Associations between PD-1 expression, parasitemia, and inflammatory markers were limited and subset-specific.

**Conclusions:**

PD-1 expression on B cells during malaria differed according to exposure history and B-cell differentiation state. Broad PD-1 induction during acute malaria was observed only in individuals living in a malaria-endemic setting, and PD-1 expression was enriched among *P. falciparum*-binding B cells. Together, these findings support a potential role for checkpoint pathways in humoral immune regulation during repeated malaria infections.

**Supplementary Information:**

The online version contains supplementary material available at 10.1186/s12866-026-05388-8.

## Background

Malaria remains a major global health challenge, causing substantial morbidity and mortality particularly in Sub-Saharan Africa [1]. Infection with *Plasmodium falciparum* (*P. falciparum)*, the most deadly human malaria parasite, accounts for the majority of severe cases and deaths. Despite major advances in malaria control through vector management, chemoprevention, and improved diagnostics, reductions in global malaria incidence have stalled in recent years [1]. The introduction of malaria vaccines, including RTS, S/AS01 and R21/Matrix-M, represents an important advance. However, vaccine-induced protection remains partial and relatively short-lived, particularly in young children and in populations with chronic malaria exposure [2–5]. These limitations underscore the difficulty of inducing durable immunity in settings where repeated antigen exposure and immune regulation shape host responses.

In endemic regions, individuals gradually acquire immunity that protects against severe disease but does not prevent infection [6, 7]. This naturally acquired immunity is largely antibody-mediated and allows individuals to tolerate parasitemia with fewer clinical symptoms [8–13]. However, malaria-specific antibody responses often wane rapidly, particularly in early life, suggesting inefficient generation and maintenance of long-lived humoral immunity [14–16]. Accumulating evidence indicates that immune regulatory mechanisms play a central role in balancing protection and pathology during repeated malaria infections [17, 18]. Acute malaria is also associated with pronounced inflammatory responses, including elevated interferon-γ (IFN-γ) and osteopontin (OPN) levels [19, 20]. While immune regulation may help limit excessive inflammation, it may also influence the development and maintenance of durable, high-affinity antibody responses [21, 22].

B cells are central to humoral immunity through their differentiation into antibody-secreting plasma cells and memory B cells (MBCs). In malaria, chronic exposure is associated with substantial remodeling of the B-cell compartment, including expansion of atypical memory B cells (atMBCs) [23–28]. These cells are characterized by altered phenotypes and enrichment of inhibitory receptors and have been linked to chronic immune stimulation in malaria and other persistent infectious conditions [29–32]. Although atMBCs are a prominent feature of malaria-exposed populations, their functional role remains debated, and the regulatory pathways shaping their development and activity in vivo are incompletely understood.

Programmed cell death protein 1 (PD-1, CD279) is an inhibitory immune checkpoint receptor that regulates cellular activation and immune homeostasis [33–35]. PD-1 is transiently induced following activation across multiple immune cell types, including T cells, B cells and natural killer cells [36]. Sustained PD-1 expression has classically been associated with impaired immune function in chronic infection and cancer [37, 38]. However, PD-1 expression is not synonymous with exhaustion. Increasing evidence suggests that PD-1 expression may also reflect immune activation, regulation, and adaptation during chronic antigen exposure [36, 39]. Experimental blockade of PD-1 signaling can enhance antimicrobial and antitumor responses but may simultaneously increase immune-mediated pathology [40], highlighting the importance of PD-1 in balancing pathogen control with protection of host tissues.

The role of PD-1 signaling in infectious diseases has also attracted increasing interest beyond oncology. In hepatitis B and HIV infection, early studies report partial restoration of pathogen-specific immune responses following PD-1 inhibition, including reductions in circulating hepatitis B surface antigen [41, 42], and enhanced virus-specific T-cell responses [43]. Similarly, experimental models of chronic parasitic infections such as leishmaniasis demonstrate that PD-1/PD-L1 (programmed death-ligand 1) blockade can improve T-cell function and pathogen control [44, 45]. Together, these findings support a broader role for PD-1-mediated immune regulation across infectious diseases and motivate further investigation of checkpoint pathways in malaria.

Although PD-1 has been extensively studied in T cells, its role in B-cell biology remains less well defined. Experimental studies indicate that PD-1 signaling can limit B-cell receptor-mediated activation, proliferation and cytokine production, while also influencing memory B-cell differentiation and antibody production [34, 46]. Human studies further support a role for PD-1 in B-cell regulation. Individuals with inherited deficiencies in PD-1 or PD-L1 display reduced memory B-cell numbers, impaired antibody responses to pathogens, and decreased B-cell receptor diversity, indicating a B-cell-intrinsic requirement for PD-1 in effective humoral immunity [46]. However, human data on PD-1 expression and function in B cells remain limited, particularly in the context of naturally acquired infections and repeated antigen exposure.

In malaria, most studies of PD-1 have focused on T cells, where increased expression has been associated with immune regulation and reduced helper function [23, 47, 48]. Experimental models further demonstrate that PD-1 blockade can enhance T-cell help and improve humoral responses during malaria infection [49]. Emerging evidence suggests that PD-1 signaling may also directly shape B-cell responses. PD-1-expressing memory B-cell subsets are more frequent in malaria-exposed adults than in malaria-naïve individuals and controlled human malaria infection studies have reported increased PD-1 expression on memory B cells following exposure [50, 51]. However, it remains unclear how PD-1 expression during acute malaria is shaped by prior exposure history, how it differs across B-cell subsets, and whether it primarily reflects activation, regulatory adaptation, or dysfunction. In addition, it remains unresolved whether PD-1 expression is preferentially associated with *P. falciparum*-specific B cells or reflects broader antigen-independent immune activation.

To address these knowledge gaps, we characterized circulating B-cell subsets and PD-1 expression in individuals with different malaria exposure backgrounds during acute *P. falciparum* infection and in healthy controls. PD-1 expression on *P. falciparum*-specific and non-binding B cells was further assessed. By defining how PD-1 relates to B-cell subset distribution, antigen specificity, and malaria exposure history, this study aims to improve our understanding of B-cell regulation during malaria and its potential implications for the development of durable humoral immunity in endemic settings.

## Methods

### Study site and study participants

Peripheral blood mononuclear cells (PBMCs) from individuals with clinical malaria were collected between March and December 2022 as part of a prospective longitudinal study at Iganga General Hospital, located approximately 120 km east of Kampala, Uganda, as previously described [20, 52]. This was an observational cohort study. Malaria transmission in the study area occurred throughout the year, with transmission peaks following the two annual rainy seasons. Adults and children presenting with fever or a history of fever within the preceding 24 h and a confirmed diagnosis of malaria were eligible for inclusion. Infants under six months of age, individuals with chronic medical conditions such as sickle cell disease or HIV infection, and individuals who had received a recent blood transfusion within the previous three months were excluded. Clinical and demographic data, including age and sex, were collected at enrollment. Follow-up visits were conducted approximately 42 days after enrollment, either at participants’ homes or during clinic revisits.

PBMCs from adult male blood donors residing in Kampala were included as malaria-exposed immune controls. These individuals were asymptomatic at the time of sampling, reported feeling well, and had no evidence of acute illness at enrollment. Due to long-term residency in a malaria-endemic area, they were presumed to have experienced continuous malaria exposure [53]. PCR screening for malaria infection was performed to assess subpatent parasitemia; one asymptomatic PCR-positive individual was retained in the study.

Between 2018 and 2022, adults with acute imported malaria were consecutively enrolled at the Department of Infectious Diseases, Skåne University Hospital, Sweden. This cohort included individuals with different malaria exposure backgrounds. Some participants were considered malaria-naïve travelers, whereas others originated from malaria-endemic regions but had resided in Sweden for prolonged periods and were therefore presumed to have reduced malaria exposure and waning immunity. Demographic information, including age, sex, country of origin, and length of residency in Sweden, was collected. In addition, healthy adult male and female individuals residing in Sweden with no known history of malaria exposure, recent febrile illness, or current symptoms of infection were included as malaria-naïve controls.

Ethical approval for the study was obtained from relevant review boards in Uganda and Sweden, and written informed consent was obtained from all participants or their legal guardians.

### Malaria diagnostics

In both Uganda and Sweden, blood samples for malaria diagnostics were collected prior to the initiation of anti-malarial treatment. Malaria infection was initially identified using a malaria rapid diagnostic test (RDT) (in Uganda: RightSign Malaria Ag HRPII/Pan Plasmodium Aldolase RDT; Hangzhou Biotest Biotech Co., Ltd., China; in Sweden: Carestart Malaria HRP2/pLDH Pf/Pan Combo test) and subsequently confirmed by microscopy or loop-mediated isothermal amplification (LAMP) (HumaTurb C + A Loopamp™ Malaria Pan Detection kit, Human Diagnostics Worldwide, Wiesbaden, Germany).

Thick blood smears were prepared according to WHO standard protocols and examined by light microscopy [54]. Parasite densities were quantified by counting parasites per 200 white blood cells (WBCs) and calculated according to WHO guidelines.

In Sweden, initial diagnostic testing was performed using RDTs or LAMP, depending on local practice, followed by confirmation using light microscopy of Giemsa-stained thick and thin blood smears. Parasite identification and quantification were performed at the Department of Infectious Diseases and Clinical Microbiology at Skåne University Hospital in Lund and Malmö.

### Sample processing and PBMC isolation

Ugandan blood samples (5 mL from adults, 2 mL from children) were collected into ethylenediaminetetraacetic acid (EDTA)-coated tubes at enrollment and day 42 post-infection. Samples were transported in insulated transport containers at ambient temperature to the Biomedical Cross-Cutting Laboratory at Makerere University, where PBMCs and plasma were isolated and cryopreserved within 6 h of collection. PBMCs were isolated using density gradient centrifugation with Ficoll-Hypaque (GE Healthcare Bio-Sciences AB, Sweden). After dilution in an equal volume of Dulbecco’s phosphate-buffered saline (DPBS; Life Technologies, Stockholm, Sweden), samples were layered over Ficoll and centrifuged at 400 × g for 30 min at room temperature. Following centrifugation, plasma was collected and stored at -80 °C. The PBMC layer was collected and washed twice with DPBS (300–400 × g, 15 min per wash) to remove residual Ficoll and platelets before resuspension in 1 mL RPMI 1640 medium (Sigma, St. Louis, MO). Cells were stained with 0.4% (w/v) trypan blue and counted using a Neubauer chamber. PBMCs were subsequently cryopreserved in liquid nitrogen at a concentration of 10⁷ cells/mL in 90% heat-inactivated fetal bovine serum (Sigma, St. Louis, MO) and 10% (v/v) dimethyl sulfoxide (DMSO; Sigma, St. Louis, MO), following established protocols.

In Sweden, venous blood samples were collected into EDTA tubes and transported to the research laboratory at Skåne University Hospital in Lund for processing. Sample collection occurred between 4 and 24 h after the initial dose of anti-malarial treatment. PBMCs and plasma were subsequently isolated using LeukoSep (Greiner Bio-One, Kremsmünster, Austria) and PluriMate II (pluriSelect Life Science, Leipzig, Germany) tubes with pre-filled separation medium, according to the manufacturers’ instructions. Samples were divided into aliquots and cryopreserved in liquid nitrogen for future analysis.

### *P. falciparum* culture and purification

*P. falciparum* 3D7 strain were cultured in vitro at 4% hematocrit in O+ RBCs. The culture medium consisted of RPMI 1640-HEPES (Gibco) supplemented with 1% Albumax II (Gibco), 200 µg/mL hypoxanthine (Sigma), 25 µg/mL gentamycin (Gibco), and 5 mM L-glutamine (Gibco). Cultures were maintained at 37 °C in candle-light boxes as previously described [55, 56]. Parasites were synchronized using D-sorbitol, and late trophozoite/early schizont stages were enriched magnetically using MACS columns (Miltenyi Biotec). Enriched early schizont parasites (> 90% parasitemia) were cultured for an additional 6–12 h before pelleting at 2400 rpm for 3 min and washing three times with PBS. Final pellets were resuspended in 50–100 µL of PBS.

### Conjugation of carboxyl Qdots to *P. falciparum* ghost-infected red blood cells (GiRBCs)

The attachment of Qdots to GiRBCs has been described in detail elsewhere [57]. Briefly, GiRBCs were generated by streptolysin O (Sigma) treatment, after which 225 µg of infected ghost cells were conjugated with 2 nmol carboxyl Qdot (35 µL) using freshly prepared 10 mg/mL N-ethyl-N-dimethylaminopropyl-carbodiimide. The resulting conjugate was diluted 1:10 in 10 mM Borate buffer (pH 7.4) and stored at 4 °C protected from light until use.

### Immunophenotyping of specific B-cell subsets including *P. falciparum*-specific B cells

Cryopreserved PBMCs were thawed in a 37 °C water bath for 1–5 min. Cells were transferred to 8 mL of pre-warmed complete RPMI supplemented with 10% FBS in a 15 mL tube, and cryovials were rinsed with 1–2 mL of warm media. The suspension was centrifuged at 330 × g for 5–10 min at room temperature, the supernatant was discarded, and PBMCs were resuspended in 1 mL of complete RPMI. Following thawing, samples were rested for up to 1 h at 37 °C and 5% CO₂ before washing with 10 mL PBS and centrifugation at 330 × g for 10 min. The cell pellet was resuspended in 100 µL of flow buffer at 1 × 10⁶ cells/mL and transferred to Eppendorf tubes.

Cells were incubated with 25 µL of Carboxyl Qdot-GiRBC conjugate on ice for 30 min, washed at 400 × g for 5 min at 4 °C, and resuspended in 100 µL of flow buffer. Staining was performed using labelled mAbs against lymphocyte markers consisting of: IgD-V500 (clone IA6-2), CD27-PE-CF594 (clone M-T271), PD-1-PE-Cy7 (clone EH12.1), CD21-APC (clone B-ly4), IgM-FITC (clone G20-127), CD19-BUV 395 (SJ25C1), IgG-BUV496 (clone G18-145), CD38-APC-H7 (clone HB7), followed by two washes with 300 µL of ice-cold FACS buffer (PBS supplemented with 0.5% BSA and 2 mM EDTA). Cells were subsequently resuspended in 300 µL of ice-cold FACS buffer, and 1 µg/mL of viability dye 7-AAD was added approximately 20 min before analysis.

Stained cells were analyzed on an LSR Fortessa X-20 flow cytometer (BD Biosciences) equipped with violet (405 nm), blue (488 nm), yellow-green (561 nm), and red (640 nm) lasers, enabling detection of 17 fluorescent parameters and two scatter parameters at the FACS Core Facility, Lund Stem Cell Center, Lund University. Compensation was performed using single-stained compensation controls, and fluorescence minus one (FMO) controls were used to support gate placement and definition of positive populations. Gating was standardized across all samples using template-based gating strategies; however, minor manual adjustments were occasionally required due to variability in sample quality and population distribution between samples. Acquired data were further analyzed using FlowJo v10.10.0 (BD Biosciences).

B-cell subsets were defined as: plasmablasts (CD19⁺ CD21⁻ CD27⁺ CD38⁺), classical memory B cells (clMBCs; CD19⁺ CD21⁺ CD27⁺), activated memory B cells (actMBCs; CD19⁺ CD21⁻ CD27⁺ CD38⁻), transitional/mature-naïve B cells (CD19⁺ CD21⁺ CD27⁻), and atypical memory B cells (atMBCs; CD19⁺ CD21⁻ CD27⁻). Memory B cells were further categorized according to immunoglobulin expression into IgD⁺, IgG⁺, and IgM⁺ subsets. B-cell subsets were additionally analyzed for PD-1 expression and *P. falciparum* reactivity. The gating strategy and subset definitions were based on previously published methods [58–60]. The flow cytometric gating strategy used to define B-cell subsets is presented in Supplementary Figure S1.

### Statistical analysis

Data were analyzed using GraphPad Prism. The Wilcoxon signed-rank test was used for paired within-group comparisons, and the Mann-Whitney U test for unpaired between-group comparisons. Multiple independent group comparisons were performed using the Kruskal-Wallis test followed by Dunn’s multiple comparisons test. For paired comparisons involving more than two related variables, the Friedman test followed by Dunn’s multiple comparisons test was applied. Correlations between PD-1 expression, parasitemia, OPN and IFN-γ were assessed using Spearman’s rank correlation.

## Results

### Study population

PBMCs from 64 individuals with sufficient material for flow cytometric analysis were included in the study. Participants represented a range of malaria exposure histories and infection statuses. Acute malaria cases included 15 Ugandan participants (UG acute malaria) and 13 adults with imported malaria diagnosed in Sweden (SWE acute malaria). All acute malaria cases included in this study were classified as uncomplicated malaria, and none fulfilled the WHO criteria for severe malaria according to WHO definitions [61].

Ugandan participants with acute malaria were recruited in an area with ongoing malaria transmission and were therefore presumed to have experienced repeated malaria exposure. In contrast, individuals with imported malaria diagnosed in Sweden represented a heterogeneous cohort with varying degrees of previous malaria exposure and immunity. All participants in this group resided in Sweden at the time of enrollment and did not have ongoing malaria exposure. Two individuals were born in Sweden and were considered malaria-naïve, whereas the remaining participants originated from Sub-Saharan Africa and had lived in Sweden for varying durations, ranging from recent arrival to several years of residence. Malaria infection in this group was acquired during travel, primarily in Sub-Saharan Africa and, in a minority of cases, in South-East Asia. As continuous malaria exposure was absent and detailed lifetime exposure histories were unavailable, this group was considered to represent individuals with none to low levels of naturally acquired immunity to malaria. Paired convalescent samples were available from nine Ugandan individuals approximately 42 days after acute malaria.

To assess the impact of chronic malaria exposure in the absence of acute infection, samples from 23 healthy Ugandan males residing in an endemic setting (UG malaria-exposed) were analyzed. In addition, 13 healthy Swedish individuals without known malaria exposure (SWE malaria-naïve) were included. Baseline characteristics of the study participants, including age distribution and sex, are presented in Table 1.

**Table 1:** Demographic and clinical characteristics of study participants

Characteristics	UG acute malaria (*n* = 15)	SWE acute malaria (*n* = 13)	UG malaria-exposed (*n* = 23)	SWE malaria-naïve (*n* = 13)
Age, years, median (IQR)	21 (16–24)	45 (36–52.5)	> 18	> 18
Male, n (%)	5 (33)	10 (77)	23 (100)	11 (85)
Characteristics at admission				
Parasitemia, parasites/µL, median (IQR)	3940 (1640–12210)	28 000 (12 000–40 000)	-	-

Participant characteristics are shown for individuals with acute malaria in Uganda (UG acute malaria) and Sweden (SWE acute malaria), malaria-exposed healthy adults in Uganda (UG malaria-exposed), and malaria-naïve adults in Sweden (SWE malaria-naïve). Age and parasitemia are presented as medians with interquartile ranges (IQR), where available. Age data were not recorded for the UG malaria-exposed and SWE malaria-naïve control groups; however, all individuals in these groups were adults (>18 years of age). All acute malaria cases were confirmed by microscopy

### Circulating B cell subset composition differs by malaria exposure and infection status

**Fig. 1:**
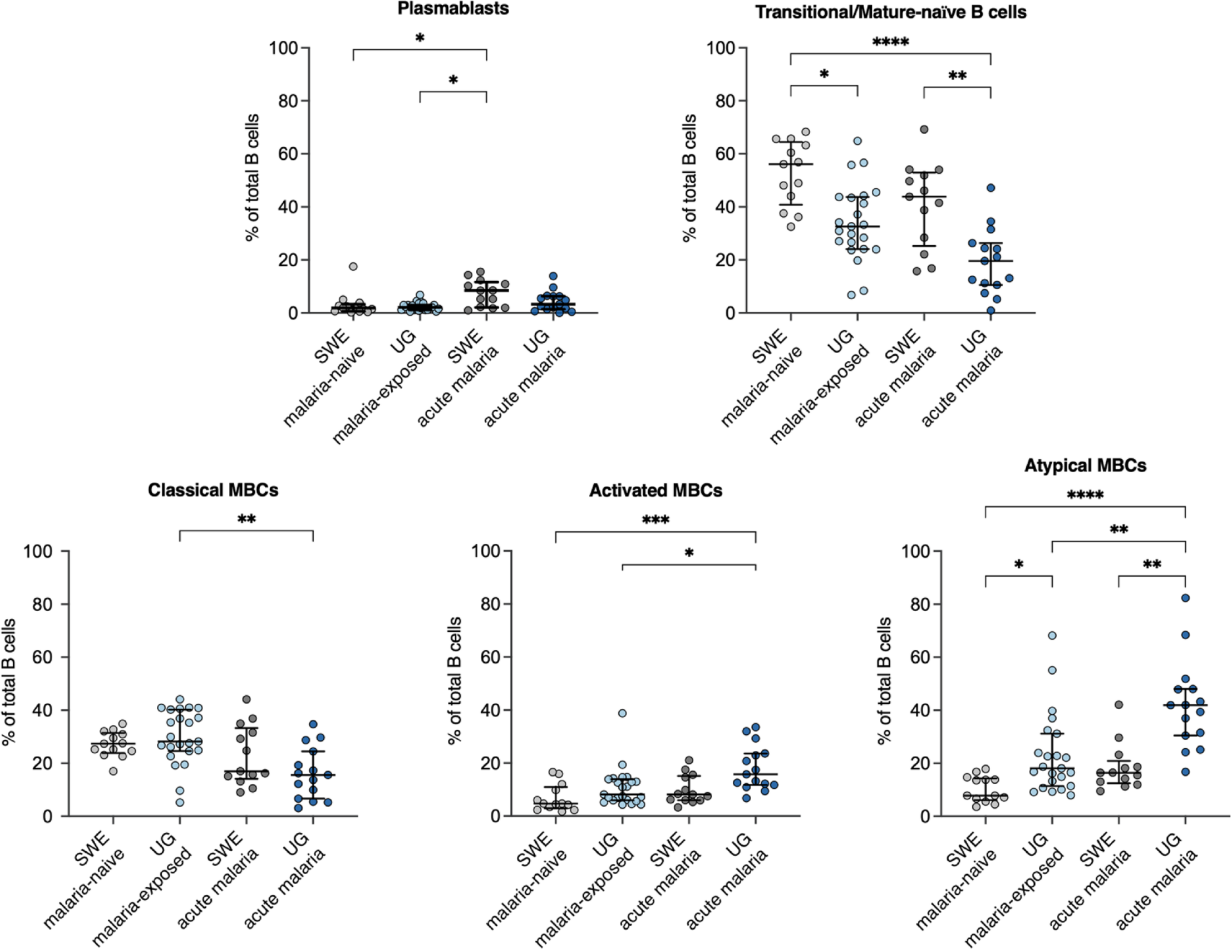
Circulating B-cell subset composition differs by malaria exposure and infection status. Frequencies of circulating B-cell subsets, including plasmablasts, transitional/mature-naïve B cells, classical memory B cells (clMBCs), activated memory B cells (actMBCs), and atypical memory B cells (atMBCs), are shown as percentages of total B cells in Swedish malaria-naïve individuals (SWE malaria-naïve, n = 13), healthy malaria-exposed Ugandan adults (UG malaria-exposed, n = 23), individuals with imported malaria in Sweden (SWE acute malaria, n = 13), and Ugandan individuals with acute malaria (UG acute malaria, n = 15). Each symbol represents one individual; horizontal lines indicate medians with interquartile ranges. Group differences were assessed using the Kruskal-Wallis test followed by Dunn’s multiple comparisons test (*p < 0.05, **p < 0.01, ***p < 0.001, ****p < 0.0001). Detailed subset frequencies are provided in Supplementary Table S1

We characterized and compared the relative proportions of circulating B-cell subsets between malaria-exposed individuals and individuals with limited malaria immunity during both acute malaria and healthy conditions. The distribution of circulating B-cell subsets differed between groups according to malaria exposure and infection status (Fig. 1). The SWE malaria-naïve cohort was characterized by a high proportion of transitional/mature-naïve B cells, whereas UG malaria-exposed adults showed a redistribution toward memory B-cell compartments, including an increased proportion of atMBCs. During acute malaria in Uganda, additional changes in the B-cell compartment were observed, including increased frequencies of actMBCs and atMBCs. In contrast, acute imported malaria was primarily associated with an expansion of plasmablasts, whereas other memory B-cell subsets showed comparatively limited changes. Detailed subset frequencies are shown in Supplementary Table S1.

. 

### PD-1 expression across major circulating B-cell subsets

**Fig. 2:**
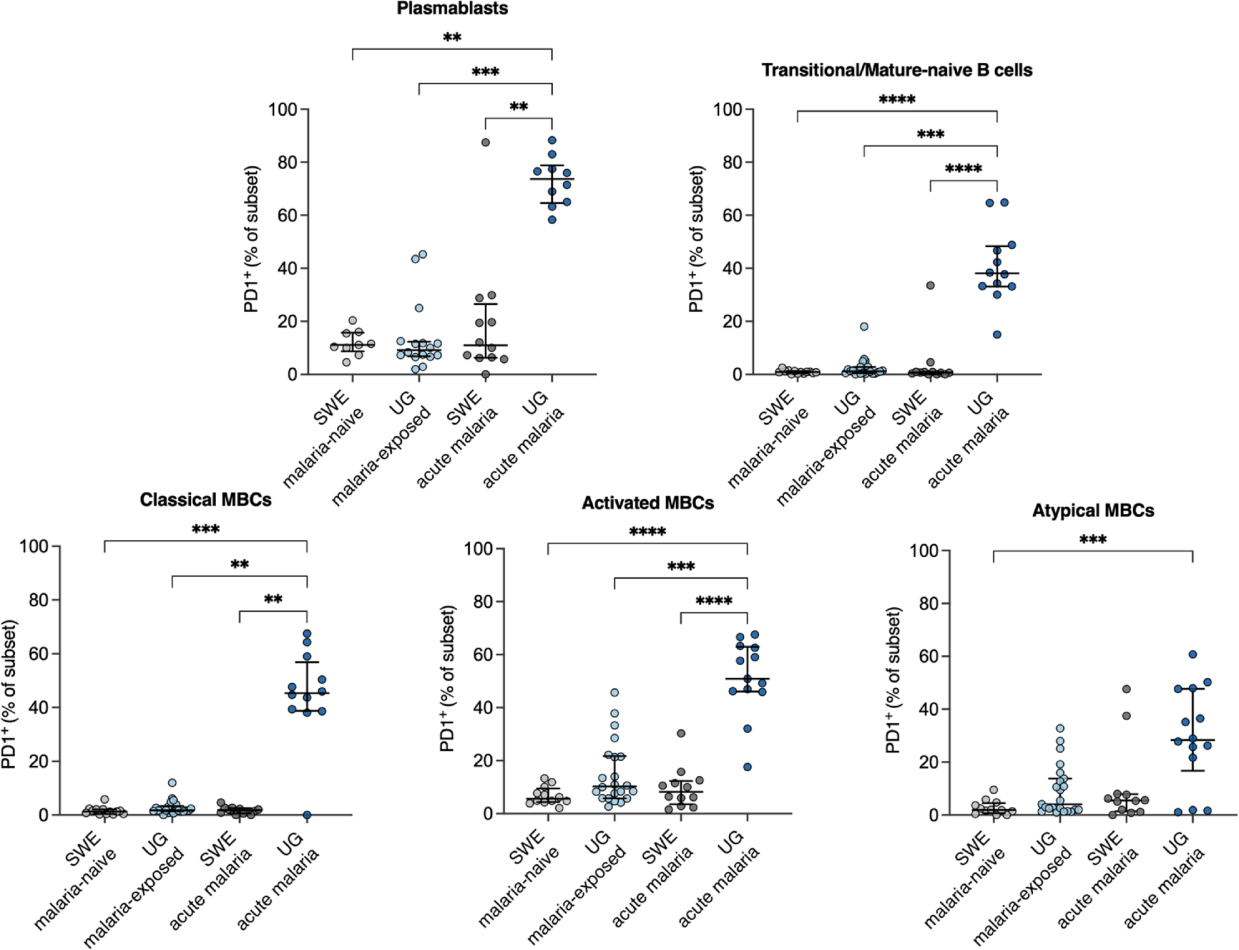
PD-1 expression across major circulating B-cell subsets differs by malaria exposure and infection status. PD-1 expression was assessed within major circulating B-cell subsets, including plasmablasts, transitional/mature-naïve B cells, classical memory B cells (clMBCs), activated memory B cells (actMBCs), and atypical memory B cells (atMBCs), in Swedish malaria-naïve individuals (SWE malaria-naïve, n = 13), healthy malaria-exposed Ugandan adults (UG malaria-exposed, n = 23), individuals with imported malaria in Sweden (SWE acute malaria, n = 13), and Ugandan individuals with acute malaria (UG acute malaria, n = 15). Data are presented as the percentage of PD-1⁺ cells within each B-cell subset. Horizontal lines indicate medians with interquartile ranges. Group differences were assessed using the Kruskal-Wallis test followed by Dunn’s multiple comparisons test (*p < 0.05, **p < 0.01, ***p < 0.001, ****p < 0.0001)

To further explore B-cell activation and regulation during malaria, PD-1 expression was assessed across major circulating B-cell subsets (Fig. 2). In UG acute malaria, PD-1 expression was increased across all analyzed B-cell subsets, including plasmablasts, transitional/mature-naïve, and multiple memory B-cell populations. In contrast, this broad increase was not observed in SWE acute malaria, indicating that widespread PD-1 upregulation during acute infection may be associated with prior malaria exposure. Although PD-1 expression was generally higher in UG acute malaria, differences between individual B-cell subsets were also observed. PD-1 expression on atypical MBCs varied less between groups but remained lower in SWE malaria-naïve individuals compared with UG acute malaria.

### PD-1 expression within activated and classical memory B-cell subsets

**Fig. 3:**
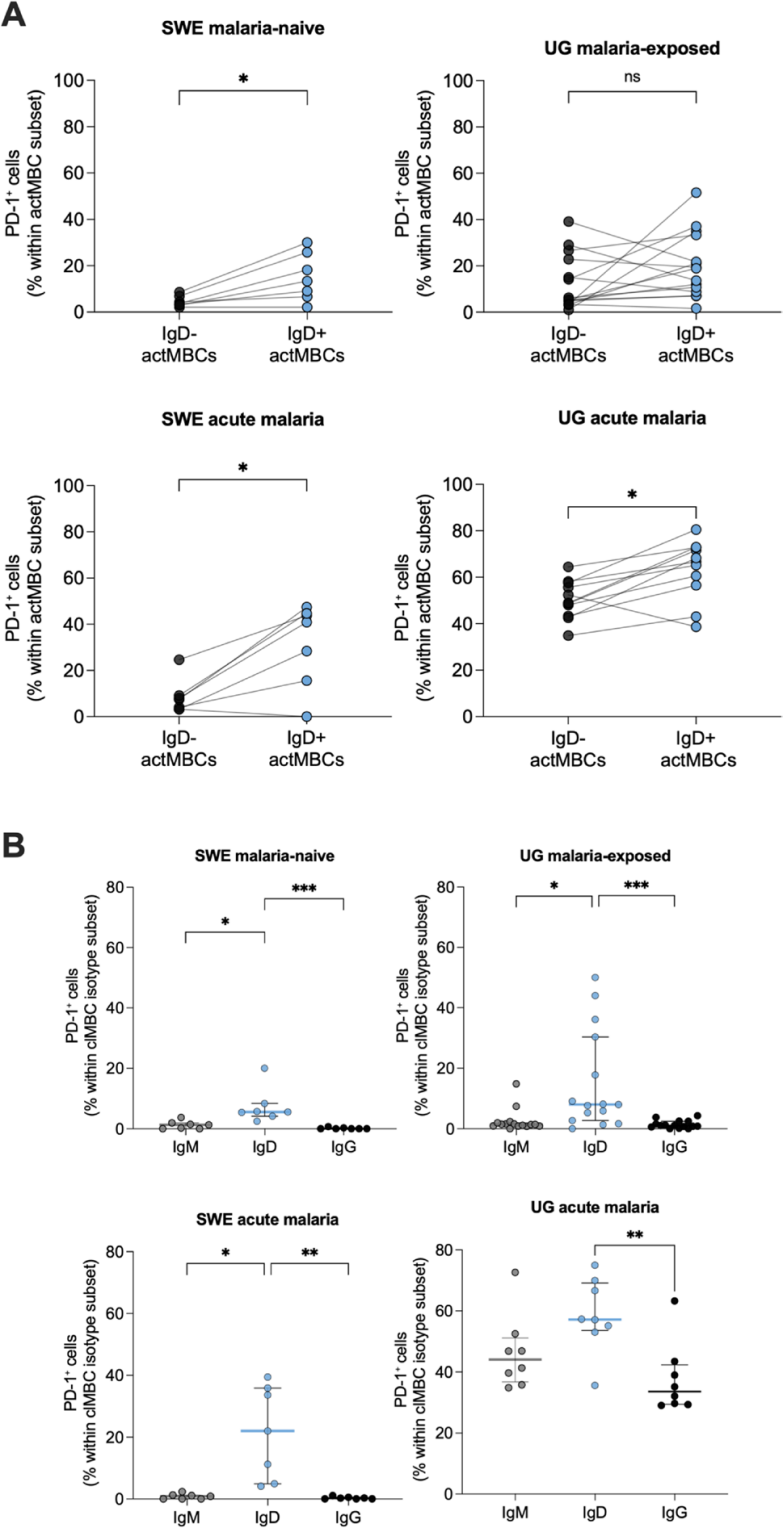
PD-1 expression differs across IgD-defined actMBCs and isotype-defined clMBC subsets. **A** Paired comparison of PD-1 expression within non-class-switched (IgD⁺) and class-switched (IgD⁻) activated memory B cells (actMBCs) in Swedish malaria-naïve individuals (SWE malaria-naïve), malaria-exposed Ugandan adults (UG malaria-exposed), Swedish imported malaria cases (SWE acute malaria), and Ugandan acute malaria cases (UG acute malaria). Each pair of points represents IgD⁺ and IgD⁻ actMBCs from the same individual; lines connect paired samples. PD-1 expression is presented as the percentage of PD-1⁺ cells within each actMBC subset. **B** PD-1 expression across isotype-defined classical memory B-cell (clMBC) subsets (IgM⁺, IgD⁺ and IgG⁺) within each cohort. PD-1 expression is presented as the percentage of PD-1⁺ cells within each clMBC subset. Each symbol represents one individual; horizontal lines indicate the medians with interquartile ranges. Statistical significance in panel A was assessed using the Wilcoxon matched-pairs signed-rank test. In panel **B**, group differences were assessed using Friedman’s test followed by Dunn’s multiple comparisons test. *p < 0.05, **p < 0.01, ***p < 0.001

Given the increased frequency of actMBCs in UG acute malaria, we next assessed whether this expansion was associated with differences in actMBC phenotype. ActMBCs were stratified into non-class-switched (IgD⁺) and class-switched (IgD⁻) populations. The proportion of IgD⁺ actMBCs was higher in UG acute malaria and UG malaria-exposed adults compared with SWE acute malaria (Supplementary Figure S2A). PD-1 expression was subsequently examined within IgD-defined actMBC subsets using paired within-individual comparisons (Fig. 3A). PD-1⁺ frequencies were higher in IgD⁺ than in IgD⁻ actMBCs in SWE malaria-naïve individuals, SWE acute malaria cases, and UG acute malaria cases. In contrast, no difference in PD-1 expression between IgD-defined actMBC subsets was observed in healthy malaria-exposed Ugandan adults.

To determine whether malaria exposure and acute infection also influenced the classical memory B-cell compartment, clMBCs were stratified according to immunoglobulin isotype and expressed as proportions of total clMBCs. IgM⁺ clMBCs were more frequent in SWE malaria-naïve individuals, whereas IgD⁺ clMBCs were enriched in UG acute malaria. No differences were observed for IgG⁺ clMBCs (Supplementary Figure S2B).

PD-1 expression across isotype-defined clMBC subsets showed preferential association with non-class-switched populations. PD-1⁺ frequencies were higher on IgD⁺ clMBCs than on IgG⁺ clMBCs across all cohorts. In addition, PD-1 frequencies on IgD⁺ clMBCs were higher than on IgM⁺ clMBCs in SWE malaria-naïve individuals, UG malaria-exposed adults, and SWE acute malaria cases (Fig. 3B).

**Fig. 4:**
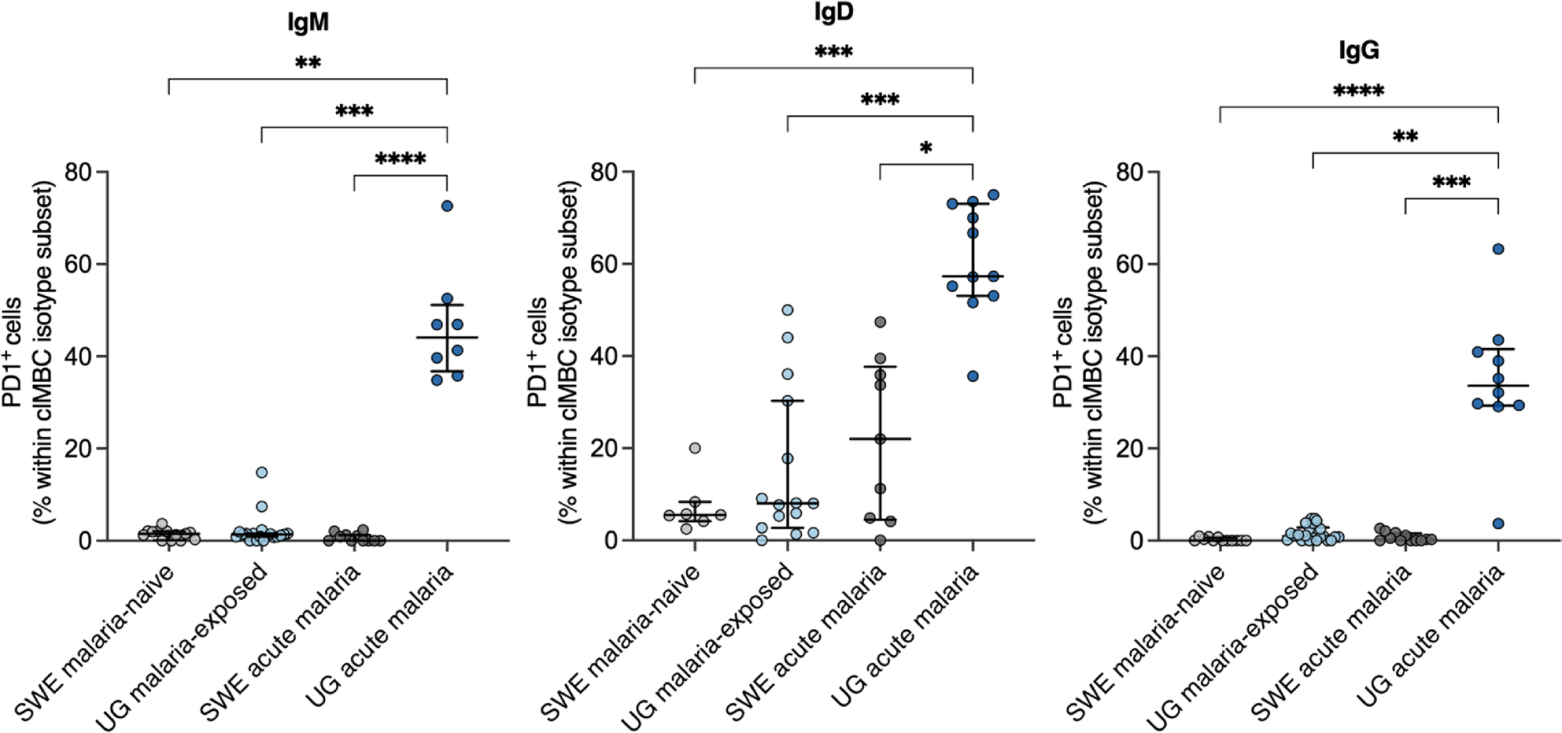
PD-1 expression across immunoglobulin-defined classical memory B cells differs between cohorts. PD-1 expression within IgM⁺, IgD⁺, and IgG⁺ classical memory B cells (clMBCs) subsets is shown for Swedish malaria-naïve individuals (SWE malaria-naïve), malaria-exposed Ugandan adults (UG malaria-exposed), Swedish imported malaria cases (SWE acute malaria), and Ugandan acute malaria cases (UG acute malaria). PD-1 expression is presented as the percentage of PD-1⁺ cells within each subset. Each symbol represents one individual; horizontal lines indicate the medians with interquartile ranges. Group differences were assessed using the Kruskal-Wallis test followed by Dunn’s multiple comparisons test. *p < 0.05, **p < 0.01, ***p < 0.001, ****p < 0.0001

To determine whether differences in PD-1 expression were consistent across immunoglobulin classes, PD-1⁺ frequencies were analyzed separately within IgM⁺, IgD⁺, and IgG⁺ B-cell subsets across all cohorts (Fig. 4). For all three isotypes, PD-1 expression was significantly higher in UG acute malaria cases compared with SWE malaria-naïve, UG malaria-exposed controls, and SWE acute malaria cases. This pattern was observed for IgM⁺, IgD⁺, and IgG⁺ B cells.

### Acute-convalescent changes in PD-1 expression and B-cell subsets (Ugandan cohort)

Paired samples from nine Ugandan individuals collected during acute malaria and approximately 42 days after treatment were analyzed to assess temporal changes in B-cell subset composition and PD-1 expression. No significant differences were observed between acute and convalescent samples in the proportions of activated, atypical, or classical MBCs, nor in transitional/mature-naïve B cells or plasmablasts. Similarly, PD-1-expressing frequencies within these subsets did not differ significantly between time points (data not shown). Together, these findings indicate that both B-cell subset distribution and PD-1 expression remained largely stable 42 days after acute malaria in individuals with continuous malaria exposure.

### PD-1 expression is enriched in *P.falciparum*-binding B cells

**Fig. 5:**
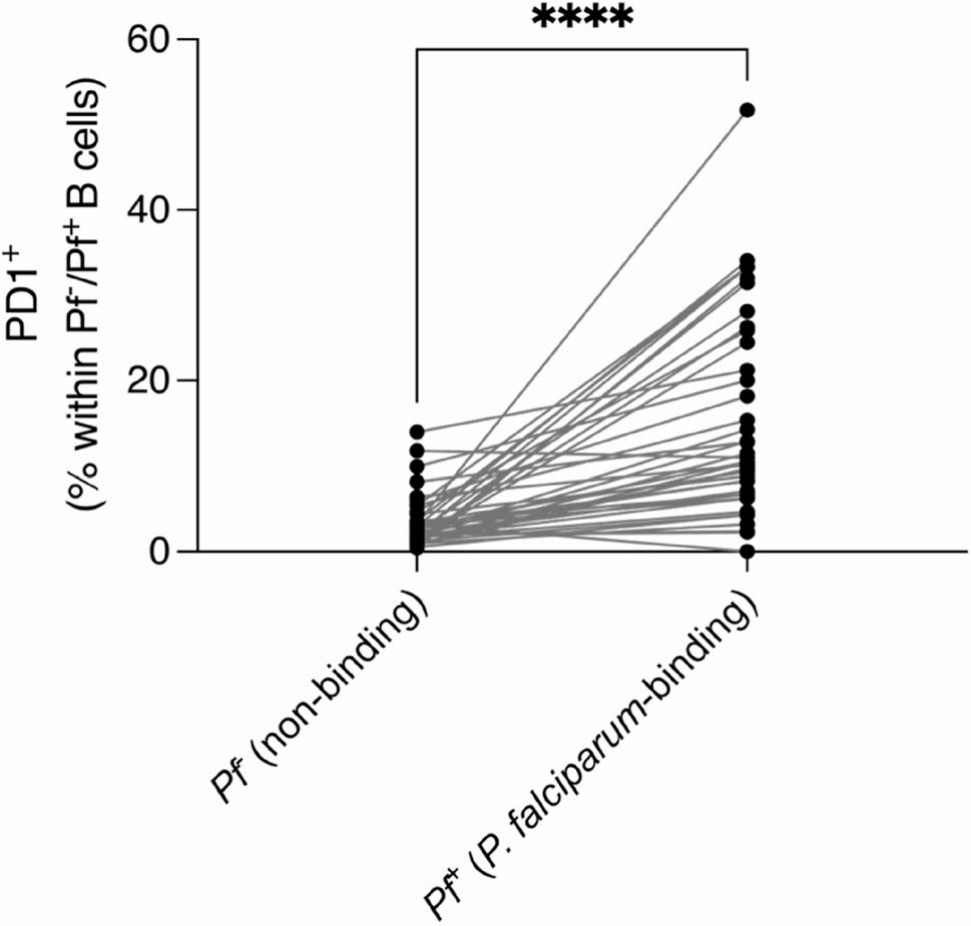
PD-1 expression on *P. falciparum-*binding and non-*P. falciparum*-binding B cells. Paired comparison of PD-1 expression within *P. falciparum*-binding (*Pf⁺*) and non-*P. falciparum*-binding (*P*f⁻*f⁻*) total B cells from the same individuals. Each point represents one individual, and lines connect paired *Pf⁻* and *Pf⁺* values. PD-1 expression is presented as the percentage of PD-1⁺ cells within the *Pf⁺* or *Pf⁻* B cell populations. Individuals with fewer than 20 *Pf⁺* events were excluded from the analysis. Statistical significance was assessed using the Wilcoxon matched-pairs signed-rank test (****p < 0.0001)

To assess whether PD-1 expression differed between *P. falciparum-*binding and non-binding B cells, PD-1⁺ frequencies were compared between *Pf⁺* and *Pf⁻* total B cells within the same individuals (Fig. 5). Participants from the SWE acute malaria, UG acute malaria, UG malaria-exposed, and UG convalescent cohorts were included. Individuals with fewer than 20 *Pf⁺* events were excluded to ensure reliable paired comparisons.

Across all included individuals, PD-1⁺ frequencies were higher in *Pf⁺* B cells than in *Pf⁻* B cells. Similar trends were observed within individual cohorts; however, these comparisons did not reach statistical significance in the UG acute malaria and convalescent groups, likely due to limited event numbers. Consequently, analyses of *P. falciparum*-binding B cells were restricted to total B cells, and subset-level analyses were not performed.

### PD-1 shows limited association with parasite burden and inflammatory markers

**Fig. 6:**
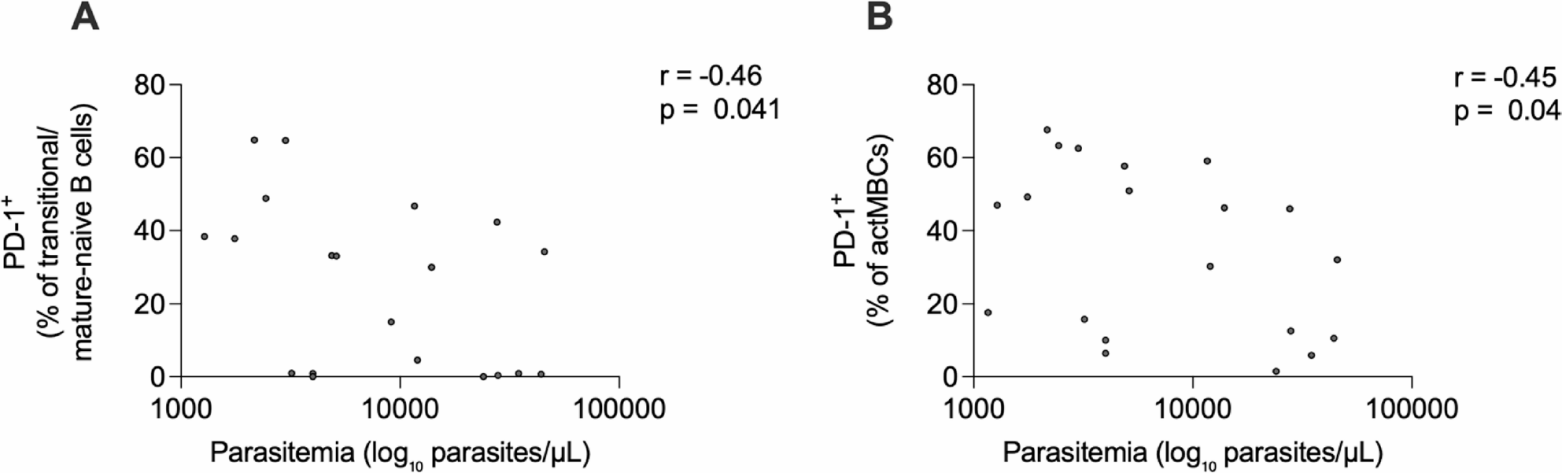
Parasitemia shows subset-specific associations with PD-1 expression on B cells. Scatter plots show the relationship between parasitemia (log₁₀ parasites/μL) and frequencies of PD-1⁺ cells within **(A)** transitional/mature-naïve B cells and **B **activated memory B cells (actMBCs). Each dot represents one individual with available paired parasitemia and PD-1 data. Correlations were assessed using Spearman’s rank correlation

To determine whether parasite burden was associated with PD-1 expression across B-cell subsets, correlations between parasitemia and frequencies of PD-1⁺ cells within major B-cell subsets were analyzed (Fig. 6). Parasitemia showed modest but significant negative correlations with PD-1 expression on transitional/mature-naïve B cells and actMBCs. In contrast, no significant associations were observed for other B-cell subsets (*p* = 0.24–0.76).

To explore whether systemic inflammatory markers were associated with PD-1 expression on B cells, correlations between plasma OPN and IFN-γ levels and frequencies of PD-1⁺ cells within selected B-cell subsets were assessed in individuals with available plasma samples. OPN showed modest, subset-specific negative associations with PD-1 expression (Supplementary Figure S3), whereas no consistent correlations were observed for IFN-γ (data not shown).

Associations between OPN or IFN-γ levels and PD-1 expression within *P. falciparum*-binding and non-parasite-binding B-cell populations were also examined. No significant correlations were observed for PD-1 expression within either *Pf⁺* or *Pf⁻* B cells (data not shown).

## Discussion

This study examined how malaria exposure history and acute infection influenced PD-1 expression across circulating human B-cell subsets in cohorts with different malaria exposure backgrounds. Acute malaria in previously exposed individuals was associated with broad upregulation of PD-1 across both naïve and memory B-cell subsets. In contrast, broad PD-1 induction was not observed in individuals with imported malaria diagnosed in Sweden. PD-1 expression also differed across immunoglobulin-defined memory B-cell subsets, with higher frequencies in IgD⁺ and IgM⁺ populations compared with IgG⁺ class-switched memory cells. In addition, PD-1 was enriched among *P. falciparum*-binding B cells. Together, these findings suggest that PD-1 expression in B cells is associated with antigen experience, B-cell differentiation, and malaria exposure history rather than nonspecific immune activation.

Consistent with previous reports, higher frequencies of atypical MBCs were observed in healthy malaria-exposed Ugandan adults compared with malaria-naïve individuals. Expansion of atypical MBCs has been described in several chronic infectious settings, including malaria, although their functional role remains incompletely understood [23, 26, 28, 62–66]. In the present study, frequencies of both atypical and activated MBCs increased further during acute malaria in previously exposed individuals. In contrast, imported malaria cases were characterized by a marked plasmablast response and relatively modest changes in memory B-cell subsets. The observed differences in B-cell subset responses align with earlier observations that B-cell responses during acute malaria differ according to prior exposure and immunity status [67, 68]. Together, these findings support the concept that repeated malaria exposure shapes the differentiation and composition of B-cell responses during subsequent infections.

Previous studies have reported increased PD-1 expression on B cells in malaria-exposed individuals compared with malaria-naïve controls, particularly within the atypical memory B cell compartment [50, 51]. In contrast, baseline PD-1 expression on circulating B-cell subsets did not differ significantly between healthy malaria-exposed and malaria-naïve individuals in the present cohort (Fig. 2). However, the sample size was limited, and subtle exposure-associated differences cannot be excluded. Variability between studies may also reflect differences in study design and cohort composition. For example, previous studies compared malaria-exposed pregnant women with malaria-naïve non-pregnant controls [50], making it difficult to distinguish effects related to malaria exposure from those associated with sex and pregnancy. A recent controlled human malaria infection study by Requena et al. further demonstrated that PD-1 expression varies depending on exposure history and experimental context [51]. Semi-immune individuals displayed higher baseline frequencies of PD-1⁺ atypical MBCs compared with malaria-naïve participants, whereas experimental infection induced increases in PD-1 expression on selected B-cell subsets, particularly in naïve or vaccinated individuals [51]. These findings differ somewhat from the present study, in which baseline PD-1 expression did not vary markedly between exposure groups. However, differences in cohort composition, cumulative malaria exposure, and flow cytometry panels may contribute to variability between studies. In addition, controlled human malaria infection differs from naturally acquired malaria in several respects, including infection dose, duration of parasitemia, and early treatment, all of which may influence the dynamics of immune activation and PD-1 expression.

One of the key findings of the present study was that broad PD-1 induction during acute malaria was primarily observed in individuals with ongoing or repeated malaria exposure. Acute *P. falciparum* malaria is associated with pronounced systemic inflammation, particularly during primary infection and in malaria-naïve individuals [69–71], whereas immune responses become modified with repeated exposure [71]. Despite substantial parasitemia, individuals with imported malaria did not show widespread PD-1 upregulation across circulating B-cell subsets, suggesting that parasite burden and systemic inflammation alone may be insufficient to explain PD-1 induction. Instead, repeated malaria exposure and recurrent inflammatory episodes may condition B-cell regulatory pathways, allowing PD-1 responses to be more readily engaged during subsequent infections.

Despite the broad increase in PD-1 during acute malaria in previously exposed individuals, regulation was not uniform across all B-cell subsets. PD-1 expression on atypical MBCs showed comparatively limited differences between groups. A significant difference was observed only between malaria-exposed individuals with acute malaria and healthy malaria-naïve controls. This limited variation suggests that PD-1 expression on atypical MBCs may be less dynamically regulated by exposure status or acute infection than PD-1 expression across other circulating B-cell subsets. Atypical MBCs accumulate following repeated malaria exposure and may persist over time in chronically exposed individuals [23, 63]. The relatively stable PD-1 expression observed on atypical MBCs during both acute infection and convalescence may therefore indicate that these populations reflect longer-term immune adaptation rather than transient activation alone. This interpretation is further supported by the observation that PD-1 expression was enriched not only in atypical MBCs but also in other antigen-experienced B-cell populations. Together, these findings support the concept that PD-1 expression may reflect immune adaptation to repeated malaria exposure rather than simple B-cell dysfunction or exhaustion. Consistent with this interpretation, malaria-exposed individuals generally presented with lower parasitemia than malaria-naïve individuals despite higher PD-1 expression across several B-cell subsets.

Within the memory B-cell compartment, PD-1 expression was closely associated with differentiation state. Across both activated and classical MBC compartments, PD-1 expression was more pronounced on non-class-switched (IgD⁺) cells than on class-switched subsets (Fig. 4), a pattern observed across multiple cohorts. As IgD⁺ memory B cells are generally considered less differentiated than class-switched memory subsets [72, 73], these findings suggest that PD-1 expression may be associated with earlier stages of B-cell differentiation rather than with more differentiated or exhausted B cells. This may point toward a role for PD-1 in activation and regulation rather than exhaustion alone. Such an interpretation is consistent with previous studies showing that PD-1 can be transiently induced during immune activation and contribute to immune regulation [74, 75].

Although PD-1 has classically been associated with exhaustion in T cells, previous studies have not identified a direct association between PD-1 expression on MBCs and malaria-specific antibody levels. This is consistent with the interpretation that PD-1 expression in B cells reflects immune activation and regulation rather than serving as a simple correlate of humoral immunity [51]. Experimental studies, primarily in mice, further indicate that PD-1 signaling can influence B-cell activation thresholds and memory differentiation [34, 76, 77], although the relevance and mechanisms of these pathways in human malaria remain to be defined.

PD-1 expression was also significantly higher on *P. falciparum-*binding B cells than on non-parasite-binding B cells in paired analyses, suggesting that PD-1 expression may be associated with antigen-experienced B-cell populations, rather than solely reflecting global immune activation or bystander inflammation. Although *Pf*-binding events occurred at low frequency and precluded subset-level analyses, increased PD-1 expression on *Pf*-specific B cells was observed consistently across exposure groups. The preferential expression of PD-1 on *P. falciparum-*binding B cells may also have implications for vaccine-induced immunity in malaria-exposed populations. Malaria vaccines such as RTS, S and R21 rely on activation and expansion of antigen-specific B cells, yet vaccine-induced antibody responses are consistently lower and less durable in individuals with prior malaria exposure [2–5]. The present findings raise the possibility that antigen-specific B cells in malaria-exposed individuals may exhibit increased PD-1 expression, which could potentially influence the magnitude or durability of vaccine-induced responses. Although PD-1 expression does not necessarily indicate dysfunction, these observations suggest that immune regulation may contribute to shaping humoral immunity following both infection and vaccination.

Associations between parasitemia, OPN, IFN-γ, and PD-1 expression were limited and varied across B-cell subsets. PD-1 patterns were also not consistently associated with systemic OPN or IFN-γ levels, in contrast to earlier studies reporting significant negative correlations between PD-1 expression on B cells and IFN-γ levels [51]. Together, the observed associations suggest that parasite burden or circulating inflammatory markers alone do not fully explain PD-1 regulation. Instead, cumulative malaria exposure may play a more prominent role.

Finally, although the sample size was limited, PD-1 expression and B-cell subset composition did not differ substantially between acute malaria and one-month convalescence. This suggests that these regulatory features persist beyond acute infection and are not solely driven by transient inflammatory responses. Alternatively, these findings may reflect a relatively stable immune state shaped by repeated exposure. Longitudinal studies over longer time periods will be required to determine the durability and functional consequences of these regulatory states.

Several limitations of this study should be considered. First, the study was designed to characterize B-cell phenotypes and regulatory marker expression and did not include functional assays to directly assess the impact of PD-1 expression on B-cell activation, differentiation, or antibody production. As such, the functional consequences of PD-1 expression on human B cells during malaria cannot be determined from these data. Second, analyses were limited to circulating peripheral blood B cells and did not capture PD-1 regulation in lymphoid tissues. Third, comparisons with previous studies are complicated by differences in flow cytometry panels and gating strategies used to define B-cell subsets. Finally, detailed information on prior malaria exposure was not available for all participants. Ugandan cohorts experienced ongoing endemic exposure, whereas individuals with imported malaria diagnosed in Sweden may have had varying degrees of prior malaria exposure and waning immunity depending on duration of residence outside endemic areas. This may have contributed to variability in immune phenotypes and PD-1 expression. In addition, differences in timing of sample collection relative to antimalarial treatment, as well as unmeasured environmental or infectious cofactors, may also have influenced B-cell subset composition and PD-1 expression across cohorts.

## Conclusions

In this study, PD-1 expression on human B cells during malaria was associated with prior malaria exposure history. Acute malaria in individuals living in a malaria-endemic setting was characterized by broad induction of PD-1 across circulating B-cell subsets, which was not observed in individuals with imported malaria who were considered to have none or low levels of malaria-specific immunity. Within the memory B-cell compartment, PD-1 frequencies were associated to differentiation state and were higher in non-class-switched memory populations. PD-1 expression was also enriched among *P. falciparum*–binding B cells. Together, these findings suggest that PD-1 responses during malaria are shaped by prior exposure, B-cell differentiation state, and antigen specificity, supporting a potential role for checkpoint pathways in humoral immune regulation during repeated malaria infections.

## Supplementary Information

Supplementary Material 1.

Supplementary Material 2.

Supplementary Material 3.

Supplementary Material 4.

## Data Availability

All results are visible in the figures, and exact datasets used and/or analyzed during the current study are available from the corresponding author on reasonable request.

## Abbreviations

PD-1: Programmed cell death protein 1
*P. f**alciparum*: *P*l*a**smodium falciparum*
WHO: World Health Organization
IFN-γ: Interferon-γ
OPN: Osteopontin
MBCs: Memory B cells
atMBCs: Atypical memory B cells
PD-L1: Programmed death-ligand 1
PBMCs: Peripheral blood mononuclear cells
RDT: Rapid diagnostic test
LAMP: Loop-mediated isothermal amplification
WBCs: White blood cells
EDTA: Ethylenediaminetetraacetic acid
DPBS: Dulbecco’s Phosphate-Buffered Saline
DMSO: Dimethyl sulfoxide
GiRBCs: Ghost-infected red blood cells
FMO: Fluorescence minus one
clMBCs: Classical memory B cells
actMBCs: Activated memory B cells
