# Supplementary material for: Malaria exposure history shapes PD-1 expression across human B-cell subsets during acute Plasmodium falciparum infection
Source: BMC Microbiol. 2026 Jul 15;26:627. doi: 10.1186/s12866-026-05388-8 (PMC13371349; doi:10.1186/s12866-026-05388-8)
Supplement: Supplementary file 1 — Supplementary Material 1. [file 12866_2026_5388_MOESM1_ESM.pdf]

B-cell subsets	SWE malaria-naïve		UG malaria-exposed		SWE acute malaria		UG acute malaria	
	%	IQR	%	IQR	%	IQR	%	IQR
**Plasmablasts**	1.9	0.6–3.3	2.1	1.1–3.0	8.4	2–11.6	3.3	1.4–6.3
**Transitional/Mature-naïve B cells**	56.1	40.8–64.5	32.6	24.1–43.7	43.9	25.3–53.0	19.6	10.5–26.3
**Classical MBCs**	27.4	23.6–31.5	28.2	24.6–40.3	17.0	14.2–33.3	15.6	6.6–24.5
**Activated MBCs**	4.8	2.9–11.0	8.2	5.9–13.9	8.2	6.0–15.1	15.8	11.8–23.6
**Atypical MBCs**	7.8	6.1–14.3	18.1	11.4–31.2	16.4	12.5–20.9	42.0	30.5–48.0

Supplementary Table S1. B-cell subpopulation median percentages calculated as proportion of total viable B cells and interquartile range in: Swedish malaria-naïve (n = 13), Ugandan malaria-exposed (n = 23), Acute malaria in Sweden (n = 13), Acute malaria in Uganda (n = 15). Statistical comparisons were performed using the Kruskal–Wallis test followed by Dunn's multiple comparisons test with adjustment for multiple testing. Significant differences are indicated in Figure 1.
