# Supplementary figures and images for: Malaria exposure history shapes PD-1 expression across human B-cell subsets during acute Plasmodium falciparum infection
Source: BMC Microbiol. 2026 Jul 15;26:627. doi: 10.1186/s12866-026-05388-8 (PMC13371349; doi:10.1186/s12866-026-05388-8)

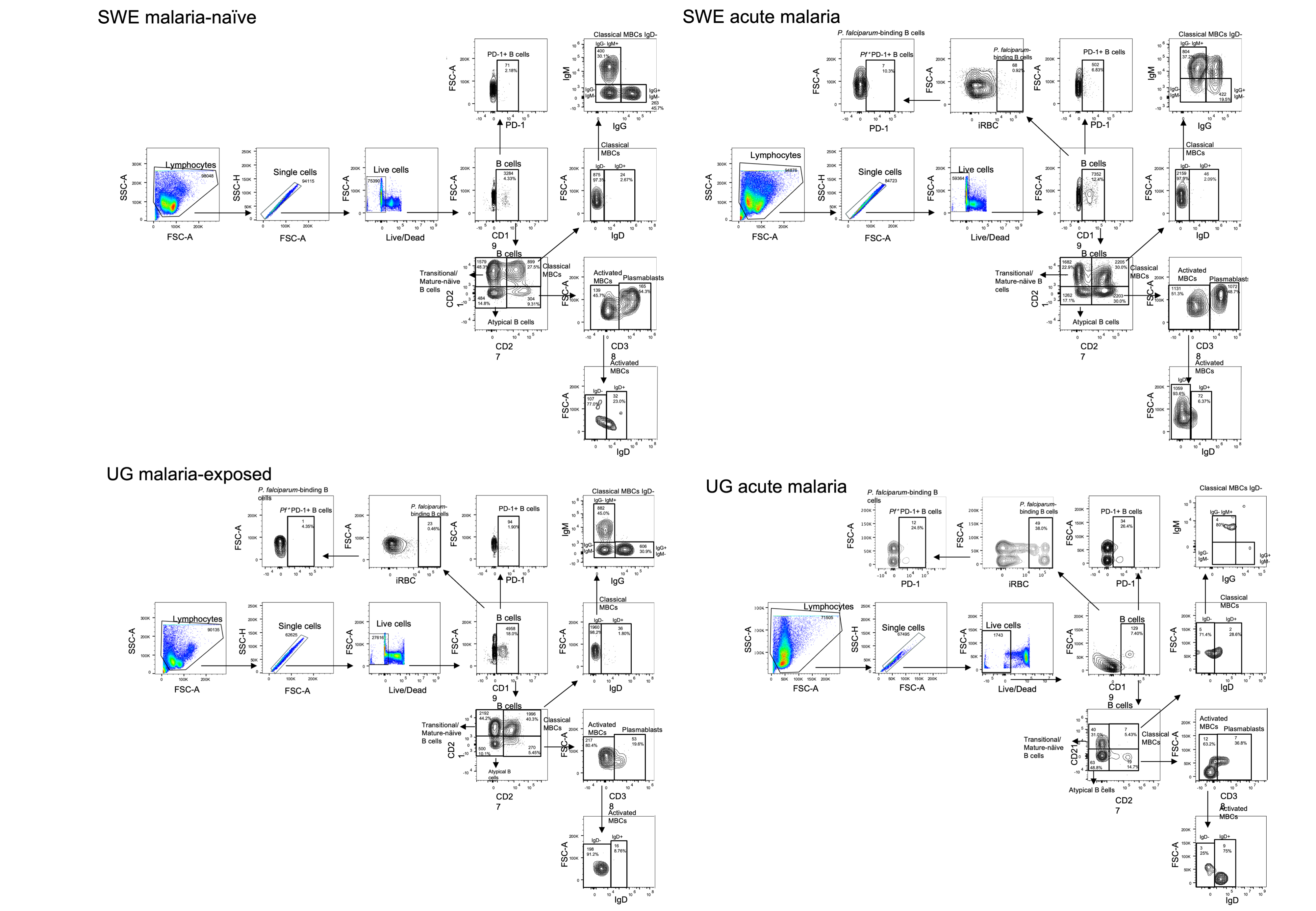

Supplement: Supplementary file 4 — Supplementary Material 4. [file 12866_2026_5388_MOESM4_ESM.png]
